# Exploring multisectoral collaboration in implementing comprehensive sexuality education framework at the provincial level in Zambia: a qualitative study

**DOI:** 10.1080/16549716.2025.2547436

**Published:** 2025-08-29

**Authors:** Malizgani Paul Chavula, Joseph Mumba Zulu, Isabel Goicolea, Anna-Karin Hurtig

**Affiliations:** a Department of Epidemiology and Global Health, Faculty of Medicine, Umeå University, Umeå, Sweden; b Department of Community and Family Medicine, School of Public Health, University of Zambia, Lusaka, Zambia; c Department of Health and Policy Management, School of Public Health, The University of Zambia, Lusaka, Zambia

**Keywords:** Capacity for joint action, collaborative governance, comprehensive sexuality education, sexual reproductive health rights, principled engagement, shared motivation, Zambia

## Abstract

**Background:**

In 2014, the Zambian government introduced the Comprehensive Sexuality Education (CSE) framework, decentralising its implementation from the national to the provincial administration. The provincial structures of the Ministries of Health and Education play an important role in providing technical, policy direction and coordination support. However, little research has focused on the role of CSE collaboration at the provincial level.

**Objectives:**

This study sought to explore multisectoral collaboration dynamics influencing the implementation of the CSE framework at the provincial level in Zambia.

**Methods:**

This qualitative study involved 29 interviews with diverse stakeholders at the provincial level such as government departments (health, education, etc.), private sector, religious and traditional leaders involved in CSE implementation. We used reflexive thematic analysis, guided by an integrative collaborative governance framework.

**Results:**

The findings were grouped under collaboration dynamics domains: principled engagement, shared motivation, and capacity for joint action. Barriers to principled engagement included provincial structures and their mandate, exclusion or sidelining of certain actors, inadequate financial transparency, and weak formal relations. Shared motivation included collective understanding of the purpose, a supportive policy environment and consensus in adapting the CSE framework. Capacity for joint action efforts included collaborative training of teachers, joint monitoring, and collaborative to address SRHR challenges.

**Conclusion:**

This study highlights challenges limiting meaningful engagement, exclusion of some actors, financial constraints, and weak coordination, which hinder collaboration. There is need for enhancing provincial leadership capacity to effectively coordinate stakeholders through enforcement of transparent resource management, collective planning, implementation and monitoring for effective CSE delivery

## PAPER CONTEXT

- **Main findings:** Provincial structures and their mandate were key aspects that facilitated principled engagement. However, barriers such as exclusion or sidelining of certain actors, inadequate financial transparency, and weak formal relations limited meaningful engagement.
- **Added knowledge:** This study highlights key factors influencing collaboration dynamics in the delivery of CSE, focusing consisting of open collaboration, collective commitment, and collaborative effort in Zambia.
- **Global health impact for policy and action:** There is greater need in Zambia to strengthen the provincial leadership capacity and power in the enforcement of transparent resource management, collective planning, equitable resource allocation, implementation and monitoring of CSE programmes.

## Background

Comprehensive sexuality education (CSE) prevents adolescents’ sexual reproductive health rights (SRHR) problems, including child marriages, unplanned pregnancies and STIs [1]. It also promotes young people’s health and well-being [1]. According to UNESCO (2017), CSE equips adolescent and young people with knowledge, skills, attitudes, and values needed to develop their self-efficacy, to foster responsible decision making and promote their health and well-being [1]. However, the implementation process of CSE remains challenging due to social, economic, political, and legal factors, in addition to the diversity of actors and multiple interests and beliefs among the actors [2]. Research has identified multisectoral collaboration as a key solution to overcoming challenges that hinder optimal adoption of the programme in school systems, particularly in low- and middle-income countries (LMICs) [2].

When it comes to CSE, there is a need for a multisectoral implementation process that involves diverse governmental actors such as health, education, social welfare, community development, private sector, and community leadership. Collaboration among these actors is fundamental to promoting the implementation of CSE as it can facilitate collective action and build trust in addressing SRHR challenges. Moreover, collaboration is also crucial because it facilitates sharing of resources and knowledge among stakeholders, which is essential for effective CSE implementation. Despite these benefits, coordination of various players involved in the implementation of CSE remains a big challenge. This complexity results in the parallel and fragmented implementation of SRHR programmes [3]. The decentralisation of CSE implementation may potentially enhance overall coordination and reduce programme fragmentation. However, unequal power relations among stakeholders can affect collaboration among actors in the implementation of CSE, which may complicate the overall governance of CSE [4].

These challenges reflect the broader complex context influencing implementation of comprehensive sexuality education in Zambia. In 2014, the Zambian government through the Ministry of Education introduced and integrated Comprehensive Sexuality Education Framework into the school systems to address SRHR issues [5]. The new Comprehensive Sexuality Education Framework (2014) replaced the outdated Reproductive Health Education Act of the 1990s, which was perceived as not being comprehensive enough. Hence, the Ministry of Education, in collaboration with stakeholders, has been scaling up the implementation of the CSE framework across the country. The availability of the enabling policy environment, including the Re-entry Policy 1997, the Education Act of 2011, and Educating Our Future, informed and authorised the development of CSE framework in Zambia. The revision process took place based on UNESCO’s international guidelines on sexuality education of 2009. The expansion of the revised framework included more sexual, reproductive, health, and rights (SRHR) themes, such as gender relations, information on contraceptive methods, sexuality, as well as values, attitudes, and self-realisation life skills [5]. Comprehensive sexuality education is not a standalone subject but has been integrated into various career subjects such as biology, social studies, home economics, integrated science, religious education, and civic education. As a way of promoting SRHR information, the target population is learners (pupils) enrolled in grades 5–12 (approximately 10–19 years old) in primary and secondary schools.

The implementation of the programme has been decentralised from the national office to the provincial administration. The provincial structures play a significant role in the provision of technical, policy direction and overseeing the collaborative efforts. There is also a notable gap in knowledge showcasing the relevance of the provincial structures in shaping collaborative implementations of the CSE programme. Studies conducted on CSE within various contexts revealed numerous issues regarding collaboration dynamics and governance affecting the implementation of CSE programmes. Despite the available evidence suggesting a supportive policy environment, contradictory perspectives on legal childhood definitions, insufficient disability inclusiveness, and contentious topics such as Lesbian, Gay, Bisexual, Transgender, Queer, Intersex, and others’ rights (LGBTQI+ rights), and abortion are some of the issues limiting optimal implementation of CSE [6]. These barriers and opportunities are potential drivers of collaboration for the implementation of the CSE programmes in school systems [5,6].

Furthermore, most studies have focused on alternative SRH education delivery through youth clubs, community settings, and the role of participatory approaches in influencing SRHR outcomes, but there is a lack of focus on the importance of collaborative governance [5,7–9]. These studies highlight the relevance of developing interventions that are culturally acceptable, yet insights into the role of collaborative governance are lacking. These studies did not document why the provincial-level actors are crucial in influencing the collaborative implementation of CSE programmes. The importance of the provincial level in facilitating the implementation of CSE is crucial, yet there is little, if any, research conducted on the role of collaboration dynamics and practices influencing the delivery of comprehensive sexuality education at a provincial level in Zambia. This study aims to fill this knowledge gap by analysing the multisectoral collaboration dynamics influencing the implementation of Comprehensive Sexuality Education Framework at the provincial level in Zambia.

Emerson et al. (2011) defines collaborative governance as the processes and structures of public policy decision making, and management that engage people constructively across the boundaries of public agencies, levels of government, and/or the public, private and civic spheres in order to carry out a public purpose that could not otherwise be accomplished [5]. In this study, the concept of stakeholders refers to individuals, groups, or organisations that have an interest or concern in a particular project, organisation, or decision [10]. The concept stakeholders also refer to actors, partners, or players representing government agencies, private sector, community, religious or traditional organisations who can influence or be affected by the project, which is CSE. Therefore, collaborative implementation entails the engagement of various actors, structures and institutions in the delivery of CSE.

**Figure 1.**
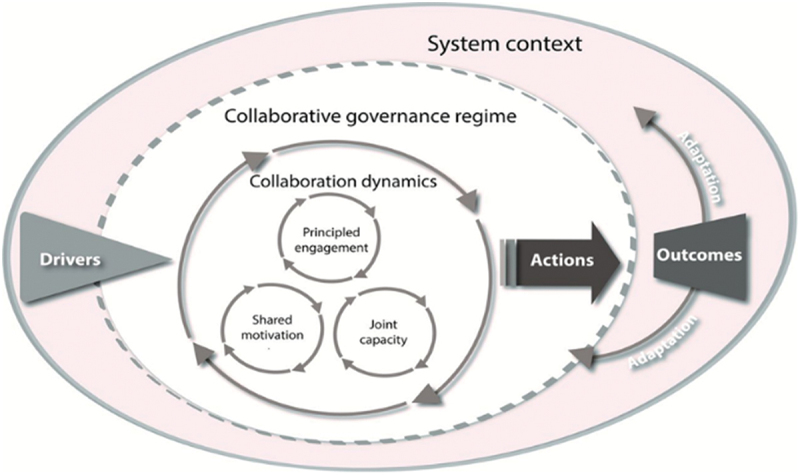
Integrative collaborative governance framework by Emerson et al. [12].

We employed an integrative framework for collaborative governance developed by Emerson and others [11] to analyse collaboration dynamics in influencing collaborative implementation of the CSE framework at the provincial level in Zambia. The framework consists of three main constructs – system context (outer layer); collaborative governance regime (meso layer); and collaborative dynamics (inner layer) – as depicted by three cyclic layers in Figure 1 [12]. In this study, the system context encompasses the national social, cultural, political and legal environment shaping implementation of CSE in Zambia. The collaborative governance regime consists of the government leadership and management of the multisectoral collaboration between stakeholders through committees from national, provincial, district and community levels for implementation of CSE in Zambia. Collaboration dynamics refers to the participation of the different stakeholders in the actual collaboration impacted by the existing system context.

We focused on exploring the collaboration dynamics layer, which include three components: principled engagement; shared motivation; and capacity for joint action. Principled engagement underscores the pivotal role of the provincial leadership in facilitating coordination, governance, agenda setting, and building consensus on controversial issues. Shared motivation involves collective mutual trust, understanding of institutional and policy mandates of CSE, collective interest in and commitment towards addressing SRHR challenges. Finally, the capacity for joint action entails engaging in collaborative activities to generate the desired outcomes. In this context, the capacity for joint action entails the actor’s ability to collectively integrate the CSE intervention within their context. Partners collectively, through regular joint meetings, mobilise resources, and collaborate in providing capacity building for teachers, collaborative monitoring, and facilitate shared learning on CSE implementation.

Furthermore, we used this framework because of its core concepts of collaboration dynamics – principled engagement, shared motivation, and capacity for joint action – that are relevant in facilitating the implementation of CSE. This framework addresses the collaborative aspects that are important for the governance of complex interventions such as CSE. Moreover, other studies have shown that collaboration dynamics significantly promote achievement outcomes of the programme [12]. In addition, measuring each element of collaborative dynamics also contributes to understanding factors crucial for enhancing optimal performance of the programme. Thus, making this framework particularly suitable for analysing multisectoral collaboration dynamics influencing the implementation of Comprehensive Sexuality Education Framework at the provincial level in Zambia.

## Methods

### Study setting

**Figure 2.**
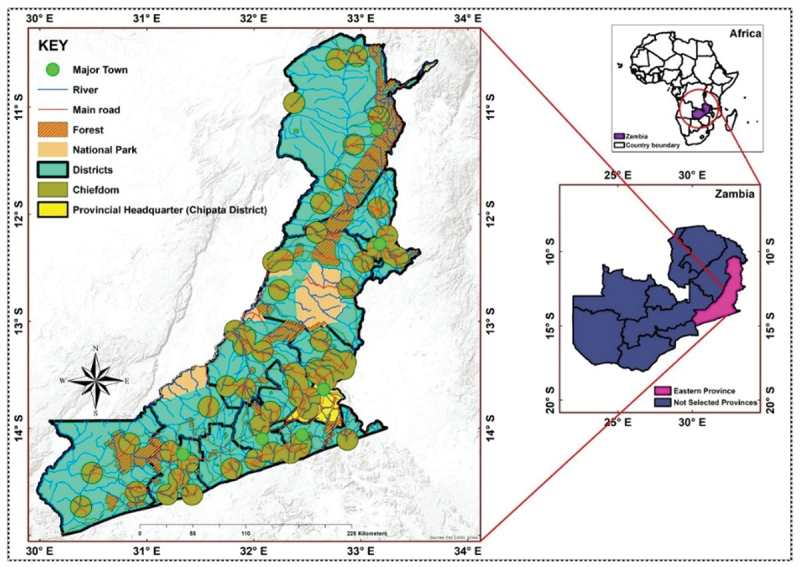
Map showing the study setting in Eastern province, Zambia.

Zambia is a low middle-income country located in central-southern Africa, subdivided into ten provinces. The governance systems and powers are decentralised to the provincial administration, which plays a significant role in providing policy implementation and service delivery. This study was conducted in Eastern Province, home of 16 districts, as illustrated in Figure 2. Currently, Eastern Province ranks second in SRHR challenges, with specifically high rates of adolescent pregnancies and marriages. Intricate social and ecological factors shape these SRHR challenges, with high rates of poverty contributing to increased unemployment and vulnerability levels, as 60% of the population lives on less than $1.90 per day [13].

In response to these various SRHR challenges, government, through the Ministries of Education and Health, are responding to SRHR challenges through the operationalisation of the Adolescent Health Strategy and a comprehensive sexuality education framework. These policies highlight the pivotal role of the Provincial Adolescent Health Technical Working Group (ADH-TWG) in the implementation, particularly through coordination, advocating, and mobilising resources for CSE. The ADH-TWG is composed of district government departments and community/school: Health; Community Development; Social Welfare; Education; Home Affairs and Internal Security (Victim Support Unit & Drug Enforcement); Youth; Sports and Arts; District Adolescent Health Coordinators; Gender; Local Government & Rural Development; Provincial AIDS/HIV Council; Chiefs and Traditional Affairs; Civil Society Organisation; and Peer educators. The Ministry of Education and Health, the secretariat, has a mandate to provide overall governance and leadership for the activities of the ADH-TWG in the province.

**Figure 3.**
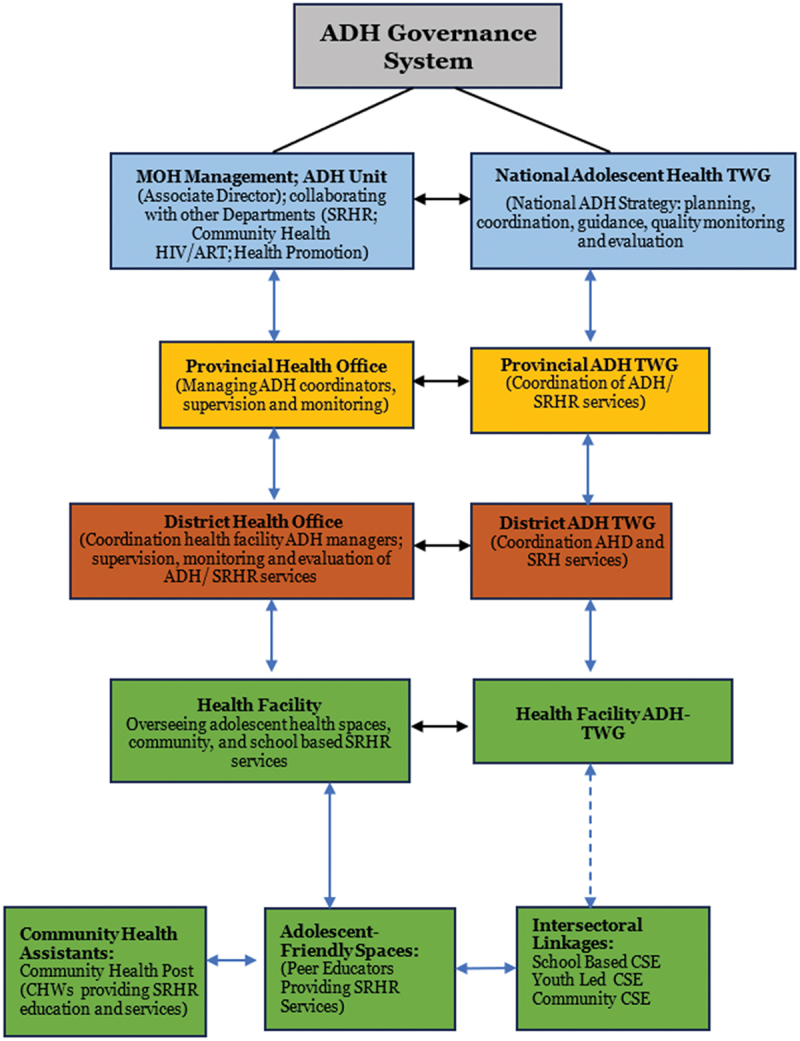
Adolescent Health system governance structure adapted from MoH [14].

To ensure effective collaborative implementation and oversight, the Provincial ADH-TWG serves as the platform for organising provincial review meetings and reporting the performance of adolescent health to the central level. Figure 3 illustrates the governance structure of adolescent health, highlighting different actors’ involvement in the delivery of CSE at various levels, including national, provincial, district and community/school. At the national level, actors provide policy, strategic direction, and overall coordination of CSE programmes. The provincial structure is responsible for overall management, coordination, and supervision in delivering CSE in the province. Meanwhile, the district-level structure plays a crucial role in the management, coordination, and supervision of adolescent health and CSE programmes by implementing partners in schools, communities, and health facilities.

### Study design

We employed qualitative case study design to explore multisectoral collaboration dynamics that influence the implementation of the Comprehensive Sexuality Education Framework in the Eastern Province of Zambia. The adoption of this approach was relevant for facilitating an in-depth understanding of various stakeholders’ experiences with provincial governance of CSE, offering valuable insights into the patterns and complexities of collaborative efforts at the provincial level [15]. This design enabled us to conduct an in-depth analysis of factors influencing the dynamics of multisectoral collaboration in CSE implementation [16].

### Data collection methods, and sampling strategy

We adopted a purposive sampling to identify national and provincial-based stakeholders involved in CSE collaboration who were eligible for interviews. The national and provincial CSE coordinators facilitated the identification and selection of these stakeholders. The selected stakeholders represented various organisations, such as government, NGOs, traditional, and religious leadership. All the participants had been involved in the implementation of the CSE programme for more than one year. Moreover, they were also listed in the Adolescent Health Strategy as members of the ADH Technical Working Group. We obtained formal permission and informed consent from the relevant departments and participants before conducting the interviews.

We continued conducting interviews until no major new themes or insights emerged from the data [17]. This approach ensured that our findings were diverse, comprehensive, and credible. We conducted a total of 29 face-to-face key informant interviews. These interviews were distributed as follows: 11 national and 18 at the provincial level (see Table 1). We held the majority of interviews in English in a safe, private space as all participants were conversant in the language. Some interviews were conducted in the local language Nyanja. Most of the participants had bachelor’s degrees, some had diplomas, and others had secondary school qualifications. The first author (MPC), with a master’s degree in public health and over 7 years’ experience in qualitative research, conducted all the interviews. To maintain an accurate record, we digitally recorded all the interviews using a voice recorder. Each interview lasted from 30 to 60 minutes. The participants were drawn from four distinct groups: interviewing actors from: government departments; (Education, Colleges, Community Development, Social Welfare, Health, Youth, National Guidance & Religious Affairs, Home Affairs (Victim Support), Traditional Affairs, Child Development, National HIV/AIDS Council), Non-Governmental Organisation, Religious Organisations, and Traditional Leaders, as illustrated in Table 1. Furthermore, we reached out to the Ministry of Health to collect the policy documents, particularly the Adolescent Health Strategy (2017–2021/20222026) to cross-check them against the actual stakeholders involved CSE implementation. This was done to gain a good understanding of the context for collaboration dynamics.

**Table 1.** Key informant interviews.

S/N	Department/Organisation	National Interviews	Provincial Interviews	Total
1	Government departments	6	9	15
2	NGOs	3	3	6
3	Religious leaders	2	4	6
4	Traditional leaders	0	2	2
Total		11	18	29

### Data analysis

The first author (MPC) and qualified research assistants transcribed verbatim the data digitally recorded using a voice recorder. We checked all the transcripts against the audio files to ensure dependability and accuracy of the data. I shared the quality-controlled transcripts, field reports, and recordings with the co-authors (JMZ, IG and AKH) for familiarisation with the material. Subsequently, I imported all the transcripts into NVivo R1, a qualitative data management and analysis software, to support the coding process. We employed abductive and retroductive inference approach in the analysis process [18]. This approach was adopted as it enables a more comprehensive analysis of complex social phenomena [18].

In this analysis process, I first read all the transcripts comprehensively to identify open codes related to collaboration dynamics affecting the implementation of CSE in Zambia. Then, I conducted focused coding through grouping common codes together. After grouping similar codes together, themes were developed to represent a higher level of interpretation. These themes were later arranged according to the predetermined constructs of collaborative dynamics, including principled engagement, shared motivation, and capacity for joint action [5]. Together with the research team (MPC, JMZ, IG and AKH), we held several meetings to discuss preliminary themes, reach consensus, and refine the analytical structure. The coding process was done based on the substantive analysis structure development in NVivo software. Additionally, we participated in a national stakeholders meeting to share and discuss the findings of this study.

Finally, to contrast the perspectives from the stakeholders involved in the implementation of CSE, we conducted a thorough review of the policy documents, particularly the Adolescent Health Strategy for 2017–2021 and 2022–26. We created Table 3 with three columns: one for listing actors specified in the policy, the second for actors identified through interviews or involved in the implementation and the third for actors excluded from the implementation process. This comparative analysis enabled us to critically analyse collaboration dynamics from both the policy and stakeholders’ perspectives.

### Reflexivity and research trustworthiness

In this study, we applied the trustworthiness criteria of Lincoln and Guba, focusing on confirmability, credibility, dependability, and transferability [19,20].

Confirmability: We ensured confirmability through thorough description of the study design, data collection methods, and analytical approaches, including member checking. As part of member checking process, we held dissemination meetings with the study participants. This process allowed us to ensure that participants recognised their perspectives in the analysis developed.

Dependability: To strengthen dependability, we ensured that we clearly described all the data collection procedures, and the characteristics of the key participants involved. For instance, interviews with government officials, NGOs, traditional, and religious leadership provided deeper insights into the context of collaboration. Additionally, we coded the material with the support from in NVivo software to maintain an audit trail of research activities, documenting verifiable steps; this contributes to enhance consistency and strengthen the trustworthiness of the results. We also adopted a flexible approach to data collection. For instance, the initially developed semi-structured interview guides were adapted during data collection to incorporate emerging contextual issues that required further exploration in subsequent interviews. This flexibility allowed for the collection of more accurate and comprehensive information on the subject.

Credibility: We strengthened credibility through conducting various interviews with different stakeholders – at both national and provincial-levels from government, NGOs, traditional and religious sectors. This facilitated gathering diverse perspectives, contributing to an in-depth understanding of the phenomenon. Furthermore, we strengthened credibility through sharing the preliminary results with the stakeholders at national level involved in the implementation of CSE.

Transferability: We strengthened transferability through clearly defining the study setting and researchers’ background, helping readers judge whether our findings can be applied to similar contexts. This detailed information allows other researchers, policymakers and practitioners to determine the applicability of the findings to comparable regions and settings. Additionally, the conceptual framework of collaborative governance was applied in both the study design and data analysis, further supporting the potential transferability beyond the specific context of Zambia’s Eastern province.

### Findings

In this section, we present key findings that influence the dynamics of multisectoral collaboration in the implementation of CSE at the provincial level. We have organised the findings around collaborative dynamics (Emerson et al. 2012) constructs – principled engagement; shared motivation; and capacity for joint action [12]. We found that provincial structures and their mandates were key factors facilitating principled engagement. However, barriers such as exclusion or sidelining of certain actors, inadequate financial transparency, and weak formal relationships limited meaningful engagement. Facilitators of shared motivation included a collective understanding of the purpose, a supportive policy environment and consensus on renaming the CSE framework to a more socially acceptable concept. Finally, factors that influenced the capacity for joint action included the collaborative training of teachers, joint monitoring, and collaborative efforts in addressing adolescents’ pregnancies and child marriages, as detailed in Table 2.

**Table 2.** Themes – collaborative dynamics constructs influencing CSE framework implementation in Eastern province.

Domains	Themes
Principled Engagement	a. Provincial structure facilitating engagement b. Lack of financial transparency and formal relationships limiting engagement c. Exclusion/sidelining of actors in the provincial engagement
Shared Motivation	a. Common understanding and supportive policy environment: addressing SRHR challenges b. Conditional shared motivation: exclusion of controversial issues
Capacity for Joint Action: Leadership Responsiveness	a. Lack of funding or resources: a driver for collaboration b. Challenges in conducting collaborative training of teachers, and monitoring c. Multisectoral collaboration: addressing pregnancy and child marriages

## Domain 1: principled engagement

Enabling factors influencing principled engagement include the provincial structure and its mandate that facilitates engagement. In contrast, inhibiting factors include the exclusion or sidelining of actors in the provincial engagement process, and a lack of financial transparency and formal relationships limiting engagement.

### Provincial structure facilitating engagement

The provincial structure, headed by the Ministries of Education and Health offices creates a platform that facilitates the engagement of relevant partners. Moreover, this structure plays a crucial role in enabling engagement of all actors through the provincial committees in setting and implementing the CSE agenda. The face-to-face engagement meetings aid planning and evaluating the province’s overall performance. This platform acts as a channel for advocating the delivery of SRHR services. Moreover, the provincial coordinators play a significant role in providing policy direction and technical support to all actors working in the province. They also oversee the operations and coordination of all provincial meetings. The coordinators engage both provincial and district-based actors for CSE collaboration. Furthermore, the provincial committees created channels and opportunities for collecting, assessing, and giving feedback on SRHR key indicators in the province. As one of the participants stated, the provincial office plays a crucial role in facilitating the engagement of stakeholders, as highlighted in the quote below:

…the Provincial education office provides guidance on policy direction regarding delivery of education in the province. Then when we get to the districts, we have officers that coordinate again, the same delivery, we work with the standards section, where the standards officers will go around schools’ district office, we also have district guidance coordinators, who are based in DEB’s offices in some districts … (24 KII, Government Official)

### Lack of financial transparency and formal relationships limiting engagement

According to the stakeholders, the provincial committees provide opportunities for planning and mobilising resources for CSE collaboration in the province. They suggested that all actors should ideally declare their resources – including finances – for the implementation of the programme. Nevertheless, a certain category of actors deliberately chooses to silently participate without disclosing their finances or resources. Because of inadequate transparency during provincial engagement meetings, resources cannot be distributed equally to districts. This is because some partners hide their budgets or implement the interventions directly. The interviews with stakeholders highlighted that lack financial transparency and formal relationships was a major factor limiting stakeholders’ engagement, as one of the participants stated:

Partners may be there, one of the challenges is that NGOs do not declare budget lines. So, when we propose to do an activity, we are at their mercy; most of the time they will not even respond. They have their project set up we are not planning together; the normal thing is that we are supposed to have an annual joint working plan. (27, KII, Government Official)

When actors with resources bypass the provincial structure and implement the activities directly at the districts-level, this hinders joint engagement in planning and the pooling of resources towards collaboration on CSE in the province; consequently, this results in unequal distribution of resources to the districts. This may also lead to uneven coverage in the delivery of CSE. For example, during the interviews, some actors discussed how direct implementation to district level creates an information gap and a vacuum for the monitoring and coordination of activities in the province.

There could be some gaps at the provincial level, some organisations would have sidelined the provincial education office, and they want to work directly with the district … But this has been difficult in terms of coordination because the provincial education office … leaves a gap in terms of knowledge by the supervising office for us to be able to say this is where we stand as a province. (01, KII, Government Official)

Furthermore, the joint planning in mobilising resources is hindered by the lack of a memorandum of understanding (MoU) between the provincial coordinators and implementing partners. This is due to poor communication between the national and provincial offices regarding actors who have the authorisation to operate in the province. In the absence of the memorandum of understanding and discussion about what and how partners can support CSE implementation and how, it becomes difficult for the provincial structure to engage the partners in a transparent, and inclusive involvement. One participant stressed the importance of having the memorandum of understanding with implementing partners as a way of promoting joint planning and resource mobilisation, as depicted in this quote:

Collaborating partners are there but we do not have a memorandum of understanding (MoUs) together. All the agreements are done by the headquarters (HQ), and NGOs work with budgets, which are decided at head offices and decisions are made from there. And the money is given with guidelines on how it is supposed to be spent. (27, KII, Government Official)

### Exclusion/sidelining of actors in the provincial engagement

The provincial technical working group is ideally supposed to include all actors, such as government departments, NGOs, and peer educators, as highlighted in Table 3. However, some actors are not aware of the existence of such committees. The participants reported that the Adolescent Health Strategy and its technical working group exclude some actors in the implementation of CSE. Our triangulation of the qualitative interviews with the policy document showed that the document outlined key actors but excluded some of them during the implementation, such as the Drug Enforcement Agency, Local Government and Rural Development, and Peer Educators, as Table 3 reveals. Moreover, other actors excluded from both the policy content and during the implementation include Child Development Department, Disability and Youth-led Organisations, as well as Religious and Traditional leaders. The quote below shows how some actors feel about the provincial engagement, citing it as undemocratic and non-inclusive.

Zambia Association of Persons Living with Disabilities, and the clergy they have been left out. We really have to inform our religious leaders of what exactly CSE is. You can find … we need… bicycles, IEC materials. So, when you look at a religious person, maybe the only thing they are going to offer us is prayer, but we do not need a prayer, we need to give information. I feel that, unconsciously, we do not deem them important because all they bring to the table is their theoretical knowledge. (18, KII, Government Official)

**Table 3.** Status of involvement of actors in provincial CSE engagement.

Actors included in the ADH policy	Actors excluded in the policy	Actors included during implementation
• Government departments: Health, Community Development, Social Welfare, Education, Home Affairs, and Internal Security (Victim Support Unit & Drug Enforcement), Youth, Sports and Arts, District Adolescent Health Coordinators, Gender, Local Government & Rural Development, Provincial AIDS/HIV Council, Chiefs and Traditional Affairs. • Private sector: Civil Society Organisation and Peer Educators.	• Government departments: Child Development, National Guidance and Religious Affairs, District Guidance and Counselling Coordinators. • Private sector: Disability and Youth-led Organisation, Religious and Traditional Leaders.	• Government departments: Health (Adolescent Health Coordinators), Community Development, Social Welfare, Education (District Guidance and Counselling Coordinators), Victim Support Unit, Youth, Sports and Arts, Gender, Provincial AIDS/HIV Council, Chiefs and Traditional Affairs. • Private sector: Civil Society Organisation.

Source: Analysis of Adolescent Health Policy − 2017/2022 and Interviews.

Finally, the coordinating team may potentially exclude, or sideline, particular actors based on their perceptions on how supportive they might be towards CSE implementation. Participants indicated that one of the reasons for excluding certain actors was to avoid resistance from those with opposing views on the agenda. Another reason participants reported was the lack of inclusion and flexibility in discussions. For example, one of the participants described how rigid and undemocratic provincial engagement meetings are, hindering flexible and open discussions. Another participant highlighted that they might be excluded from the meeting due to their strong stance on CSE, and how other stakeholders’ rigidity and intolerance toward opposing views contribute to this exclusion, as depicted in the quote:

People have different agendas and people have what I can say as positions; there are those who have a position that unless we give contraceptives to the children that’s when, and they champion just that, they don’t want to look at other alternatives. For example, religious leaders are saying we need to focus on abstinence, but then we have these other civil societies who say we just need to focus on providing contraceptives, without really looking at the submissions of other people. because they want to please funders, they want the funders … they may have hidden agendas, because if we are talking about homosexuality in in the country there are people who would come in the name of talking about life skills and health education…. (KII, 12)

The exclusion of some actors from the implementation of CSE process could have contributed to misconceptions and their lack of trust in the programme, particularly in relation that it is perceived to include controversial topics. Some participants narrated how this lack of engagement fuels misunderstandings that could have been reduced through more inclusive collaboration.

## Domain 2: shared motivation

In addition to factors shaping principled engagement, this section highlights factors that influence shared motivation. These include mutual understanding of the purpose, supportive policy environment, and conditional shared motivation shaped by the exclusion of contentious topics such as LGBTQ+ rights, abortion, and contraceptive delivery.

### Common understanding and supportive policy environment: addressing SRHR challenges

The actors are motivated to collaborate in the implementation of CSE because of a collective understanding of CSE goals, and their commitment to international and local policy guidelines. The respondents narrated that SRHR challenges, such as adolescent pregnancies, marriages, and STIs, require a collective response to address them. They indicated that comprehensive sexuality education framework covers values, attitudes, relationships, culture, society, human development, sexual behaviour, and SRH knowledge crucial to promoting sexual health and well-being. Taking part in providing CSE in schools could contribute to a reduction in alarmingly high rates of adolescent pregnancies, marriages, and STIs. The key informant interview revealed that:

The goal is to reduce teen pregnancies, marriages, and high infection rates STIs, and HIV. The purpose is to expose risk factors to learners and to help our youths make informed decisions. (29, KII, Religious Organisation)

Furthermore, the actor’s appreciation of the importance of the global health agenda on SRHR acted as another factor contributing to collective understanding in addressing challenges including child marriages and pregnancies. One of the interviewees stressed the importance of collaborating on the programme, noting that it contributes to achieving the international SRHR targets as illustrated in the following quote:

The country we are in is a global village and also subscribe to global international obligations; for example, East and Southern Region commitment … the ministries of education and health appreciate that young people are facing a few challenges regarding their adolescent sexual reproductive health, hence one of the solutions offered is the revision of CSE as well as the revision of adolescent sexual health services, and hence the partnership of the MoGE and MoH. (01, KII, Education)

Finally, participants considered a supportive policy environment as another motivating factor for collaboration. The policy environment provides directions and gives authority for key actors to act collectively to respond to SRHR needs. The participants highlighted that policy gives the legal mandate to carry out activities:

Everything that we do as a ministry is guided by policy, policy is what provides the direction for any intervention that must be done, then you are within the law and that becomes a legal framework before you do any intervention. (01, KII, Education)

### Conditional shared motivation: exclusion of controversial issues

Shared motivation is critical in building trust among actors who might initially have been opposed to CSE collaboration. At the provincial level, implementing CSE requires a collective understanding of the agenda and purpose of CSE. Reaching this collective understanding may be challenging because of different social, cultural, and religious orientations toward addressing SRHR challenges. Currently, there is a sustained exclusion of topics considered controversial, such as LGBTQ+ rights, abortion, and delivery of contraceptives in schools. Such exclusion of topics acts as a catalyst or driver for collaboration. However, some actors remain reluctant and opposed to participating due to their perception that the curriculum includes these topics, which they believe should not be included. Therefore, to reduce this resistance towards CSE implementation in schools, the participants noted that in trying to engage stakeholders it was crucial to clarify what is included and excluded in the CSE curriculum. This approach was perceived as facilitating trust building; it encourages and brings on board hesitant actors to become involved in the collaboration of CSE.

People have different agenda, and people have what I can say as positions … that unless we give contraceptives to the children that is when … , they do not want to look at other alternatives. For example, religious leaders are saying, we need to focus on abstinence, but then we have these other civil societies who say we just need to focus on providing contraceptives, without really looking at the submissions of other people. So, people come up with positions even in closed meetings, they just want their position to stand. (12, KII, Education)

… I know [21] may have hidden agendas, because if we are talking about homosexuality in the country, there are people who would come in the name of talking about life skills and health education or CSE as if to champion other things … please funders. (01, KII, Education)

Finally, consensus building through stakeholder engagement was crucial to achieve and align a common purpose. The name ‘CSE’ created misunderstanding and ignited resistance among stakeholders. To achieve conditional shared motivation, stakeholders revised the concept of CSE to make it more socially acceptable. The suggested name of ‘Life Skills and Health education’ was perceived as accurately aligning with its true essence, values, age-appropriate content, and social compatibility. This approach addresses actors’ concerns and promotes common shared understanding and commitment towards achieving the same goal, as depicted in the following quote:

Comprehensive sexuality education has changed to Life Skills and Health Education, awaiting cabinet approval; of course, some of the things have been returned in the curriculum but because of the submissions that were made by stakeholders, and we will not see some topics that are sexualising the children, aligning their thoughts to sex but now the focus is to help them navigate. Hence, they need life skills so much. (01, KII, Education)

## Domain 3: capacity for joint action

After examining factors influencing principled engagement and shared motivation in the implementation of CSE, this section presents factors influencing the capacity for joint action. These factors include lack of funding or resources as a driver of collaboration, organising, training teachers, monitoring, and multisectoral collaboration in addressing adolescent pregnancies and child marriages. These are further presented below.

### Lack of funding or resources: a driver for collaboration

The limited funding and resources acted as one of the drivers for collaboration. This inadequate resource availability motivated key actors to join forces in the collaboration for CSE. The different actors realised that pooling resources towards the same goal helped compensate for their inability to carry out the activities individually without external support. The participants reported that the willingness of various actors to collaborate facilitated the increased coverage of implementation, including in areas that a single entity or government programme might not have reached. For example, one of the participants stressed the importance of partner support as a means of increasing the resource base for CSE implementation.

Resources are scarce, and we may plan activities at our level and even scale down to the district level, but we may not have enough transport to take us to all parts of the province. So, the coming in of partners helps us reach places where there are not enough resources. We can communicate our plans with them. And they can pick out certain areas where we are unable to reach [22].

### Challenges in conducting collaborative training of teachers, and monitoring

The provincial leadership collaborated with other actors well-resourced to support teacher training and the monitoring of CSE activities in the province. One participant highlighted the importance of collaborative efforts by the provincial structures, colleges, NGOs and other stakeholders in the delivering of CSE training for teachers.

At provincial level we have a teacher education department which oversees Inservice training – so, all the training that we organise at provincial level. However, we also have organisations like I mentioned – many NGOs – who work with us. They also provide training but through teacher education at district level. (01, KII, Education)

In addition, the collaborative efforts between stakeholders facilitated effective, intensive training, as emphasised by one of the participants in the illustrative quote.

The college hub model is at the central point in the implementation. Teachers are trained for five full days through intensive training. We are working in partnership with…, a civil society organisation. Then we also have staff from the Ministry of Health, and education (provincial office) staff from the college and funding partners. They can monitor and support. (25, KII, Government Official)

Finally, the participants reported that there was inadequate joint monitoring of districts. Joint monitoring is relevant to determining actors’ compliance with the programme goals and outcomes. Moreover, it plays a crucial role in collaboratively monitoring gaps, engaging stakeholders, and ensuring the uniform implementation of the programme.

We do monitor the CSE programme and training quarterly, but there have been some challenges … We have only managed to monitor those closer to our office because resources cannot allow us to reach all the districts. CSE is integrated into career subjects, the monitoring focuses on the review of all the subjects, not specifically to CSE. It is not well monitored. (01, KII, Government Official)

### Multisectoral collaboration: addressing pregnancy and child marriages

Delivery of CSE in schools without involvement of key actors may create hurdles in addressing the increased occurrence of SRHR challenges, including girls dropping out of school due to pregnancy and marriage. To address this problem, some participants stressed the importance of strengthening the joint action between traditional leadership and the school administration in fighting against adolescent pregnancies and marriages. For example, some participants reported how traditional leaders, and the school administration are working together to combat child marriages and pregnancies through community engagement and punishing the perpetrators of GBV, including child marriages, as shown in the quote:

We have a meeting; we usually have a committee at provincial level. We meet quarterly at provincial level; education is the chair; we chair those meetings. So, we look at several issues affecting adolescence, and mostly the early marriages, early marriage issues, and teen pregnancy because those are on the agenda most of the time. (24 KII, Government Official)

Furthermore, some participants mentioned that joint action between government actors – for example, child development initiatives – facilitated the retrieval of girls from marriages. These joint actions included creating a safe environment, building safe boarding houses, and providing school support for pupils, especially girls. Interviewees stressed that their involvement was critical in preventing and ending child marriages:

The best way if you retrieve a child from an early marriage is to find a secure house for them or send a child to boarding school and there is an appropriate support system to keep the child in school. Sometimes you can hear that an early marriage is about to take place in a certain area, but you are incapacitated with no transport available. By the time you have transport you will find that it has already happened, and people have disappeared. (27 KII, Government Official)

Finally, relevant government departments, such as the police, social welfare, and child development, provide sensitisation both in schools and the community on the importance of child protection and their roles in mitigating child abuse. However, this response is fragmented and runs parallel to addressing child marriages. The competing interest hinders effective joint actions by the departments or actors involved in the collaboration. This negatively affects their joint effort in the delivery of CSE-related programmes, both in schools and communities. For example, dealing with child abuse requires available resources, and the lack of these contributes to barriers preventing victims from promptly accessing services such as health, justice, safety, social and economic support. The quote below highlights how different actors are collaborating in conducting sensitisation and providing financial empowerment as key programmes to prevent child marriage:

We have been conducting sensitisation through district committees in the Eastern Province … since this department has no offices in all districts … because we do not have the finances. … they are directly involved in curbing child marriages, but we meet a lot of challenges along the way. We retrieve a child from an early marriage and so what? We do not have anywhere to take the child, as it is in the appropriate place for their safety. (19 KII, Government Official)

## Discussion

This study analysed the multisectoral collaboration dynamics influencing the implementation of Comprehensive Sexuality Education Framework at the provincial level in Zambia. The enabling factors affecting principled engagement included the provincial structures facilitating engagement. The inhibiting factors included the exclusion or sidelining of actors in the provincial engagement, the lack of financial transparency, and formal relationships limiting engagement. The study also highlighted factors shaping shared motivation, such as striving to attain a collective understanding of the purpose and supportive policy environment, leveraging on conditional collective interests in resolving tensions, and changing the CSE name to life skills and reproductive health education. Finally, the capacity for joint action included collaboration in organising and monitoring the training of teachers and in addressing child/adolescent pregnancy and marriages.

### Drivers or factors influencing multisectoral collaboration for CSE implementation

The social, economic, political, and legal environment is a major driver for CSE implementation in Zambia. This study identified, on the one hand, that inadequate resources, including finances and materials, were factors hindering optimal delivery of the CSE programme. However, this limitation in available resources, on the other hand, also created opportunities for collaborations through the pooling of resources together for the implementation of CSE. Despite this potential for collaboration, this study revealed that some actors failed to declare their resources to the relevant authority. This poses an important challenge, since similar studies have shown that integrating multiple funding sources promotes collective ownership, responsibility, and social accountability in programme implementation [23]. We also found that limited participation and inclusion of actors in the engagement process of CSE contributed to parallel implementation and duplication of efforts. Strengthening stakeholder involvement is therefore essential for facilitating joint planning and effective implementation of CSE.

Our findings highlight a significant challenge: some key actors bypassed provincial structures and proceeded with the direct implementation of activities at the district level. This approach contributed to limited pooling of resources, which might be essential to support the provincial and district-based training and monitoring of CSE activities. This lack of transparency and financial disclosure generates mistrust, which undermines the collaborative delivery of CSE due to parallel implementation of the programme among the partners involved. We further found that lack of transparency and financial disclosure hinders optimal and meaningful collaboration and social accountability. While another study from Zambia suggested that direct implementation at the district level can enhance local actors participation and improve programme outcomes in the short term [24], our findings highlight the long-term challenges. Although actors bypassing of the provincial administration may promote local accountability and ownership, our findings show that this practice limited joint planning and potentially led to an uneven distribution of resources in the province.

The shortage of resources enhances the sense of interdependence and fosters a mutual understanding among crucial actors, motivating them to collaborate in the delivery of CSE. Furthermore, similar evidence supports the notion that when partners realise that they cannot achieve their goals independently, they turn to other organisations to foster collective action through mutual reliance [25]. While financial constraints can sometimes foster collaboration by encouraging resource-sharing among stakeholders, the absence of adequate internal funding remains a significant challenge. Limited financial support weakens programme implementation, restricts stakeholder involvement, and undermines long-term sustainability. In LMICs, inadequate funding for health programmes often leads to fragmented efforts and reduced impact, making reliance on external collaboration insufficient for ensuring stability [26,27]. Therefore, while partnerships play a vital role, securing sufficient internal funding from both central and local governments is essential for effective, coordinated, and sustainable implementation of CSE.

### The importance of provincial leadership and mandate in facilitating collaboration

Our study found that effective leadership is crucial in facilitating collaboration among provincial actors in the delivery of comprehensive sexuality education in schools. Effective leadership plays a significant role in providing overall coordination of programmes through facilitating joint planning meetings, ensuring equitable resource allocation, promoting collaborative implementation, and overseeing the monitoring of activities [13]. However, our study expands on this by illustrating that leadership is crucial not only for programme coordination but also for overcoming challenges such as difficulties in achieving shared interests, inadequate resources or funding allocation, and insufficient effective communication channels. This highlights the need for strong leadership both at the national and the provincial levels to effectively engage all actors in joint planning and collaboration in the delivery of CSE.

Furthermore, this study reported that the existing provincial leadership structures experienced challenges with coordination meetings because of powerful actors who silently refused to declare their budgets, actors who bypassed provincial structures, and the exclusion by leadership of other crucial actors, including persons with disabilities, youth groups, community, and faith-based organisations in the engagement processes. This shows the limited capacity of the established leadership to manage complex programmes such as comprehensive sexuality education. Similar studies highlight the importance of leadership skills in the facilitation, negotiation, and collaborative problem-solving, and strategic thinking to effectively manage collaborations in these complex settings [28]. Leadership is crucial in facilitating the brokering of the engagement of actors to build trust and relationships for inclusive planning, and joint action in the delivery of CSE [29]. This study found that the provincial leadership had limited capacity to effectively coordinate all the actors inclusively.

This calls for policy reform to strengthen the mandate and authority of the provincial leadership in managing and brokering the implementation of CSE. The revised policy should clearly highlight their functional responsibilities in relation to the governance of health programmes including CSE, thus empowering provincial leadership. A South African study also reported that devolving governance power to provincial authorities might increase their capacity to discipline implementation actors who fail to comply with collaboration guidelines, including transparency and financial management regulations [30]. While this strengthens their authority to enhance oversight and may potentially improve adherence to standards, it also introduces more tensions that may affect collaborative dynamics. For instance, the ability to discipline non-compliant stakeholders can reinforce the legitimacy of the CSE programme and promote a culture of accountability [31]. However, when such authority is exercised without caution and inclusiveness, it may be perceived a top-down mechanism, potentially discouraging open dialogue and mutual trust among stakeholders. However, when such authority is embedded within a supportive policy and legal environment, a balanced approach to authority and collaboration can support an enabling environment for the effective and equitable delivery of CSE in schools [32].

The inadequate communication between the national, provincial, and implementation levels hindered optimal collaboration in the delivery of SRHR services. Inadequate communication between the national and provincial structures jeopardises coordination. Evidence also suggests that lack of coordination between the health sector workers and other actors – including village leaders, communities, and women – is problematic due to the design, local context, lack of accountability of the scheme to local communities, and unequal power relations [13,33]. Therefore, increased communication through coordination meetings, both virtual and ad hoc, is critical to effectively coordinate the implementation of CSE in the provinces [34]. Similar studies on multisectoral approaches in public health reported that complex interventions like CSE a need robust communication process, which are critical for stakeholder collaboration [35].

### Challenges and enablers of multisectoral collaboration in CSE

This study also reported the complexities of joint cross-boundary collaboration in the implementation of CSE. Our findings revealed that the exclusion of controversial topics such as abortion, contraceptives, and LGBTQ rights is one of the factors that motivate partners to continue collaborating. Though this builds consensus in the implementation of CSE, wide consultations are needed to understand the effects of the sustained exclusion of such issues from the CSE framework. Similar studies also indicate that collaborative governance facilitates the engagement of actors with different interests and values, roles, resources, and relationships to deliver the required services [29,36,37]. Furthermore, a Rwandan study that analysed the content of comprehensive sexuality education for early adolescents found that the consultative process is challenging in yielding a balance between additional content on locally controversial issues and aligning with internationally recognised topics, such as sexual pleasure, orientation, desire, and modern contraceptive methods [38].

This study highlighted the controversies of the comprehensive sexuality education concept, which is associated with promoting sexual activity because SRHR, including contraceptives, are often considered as only being socially appropriate for married people. A solution to this would be to revise the term to align with the local context. Nevertheless, renaming the CSE concept might be misinterpreted as limiting it to life skills and health alone, without considering the inclusion of broader aspects of CSE such as reproductive justice and the human rights approach to accessing SRHR services. This calls for meaningful engagement to facilitate successful collaboration. It also requires actors to come together to reach a common purpose and ground to identify mutual interests, and resolve the differences with respect and honour the perspectives of all the parties involved [37,39]. Similarly, evidence also confirm that aligning CSE with the local context can enhance not only its acceptability but also its effective implementation [40]. However, other studies contradict this view, suggesting that renaming CSE could dilute its comprehensive nature, potentially excluding critical components such as reproductive justice and human rights [41].

Finally, our findings showed that multisectoral collaboration between government, community-based actors, and civil society is essential to address key aspects of CSE including adolescent pregnancies and marriages. However, our study has also reported disjointed patterns of collaboration in addressing these SRHR challenges. The government actors, civil society, and traditional leadership fought against child marriages independently, creating a vacuum for an interconnected response. Similarly, evidence from low middle-income countries found that weak governance mechanisms and fragmented implementation hinder effective multisectoral collaboration in the delivery of SRHR programmes due to lack of synergy which contributes to duplication of efforts among entities [42]. The lack of synergy and duplication has often linked to implementing partners receiving funding from international agencies who insist on independently conducting the activities [43,44]. These findings are crucial for reforming current policies to encourage joint funding or the pooling of all resources together and distribute them equally and equitably across all the provinces. Such an approach would strengthen multisectoral response to address SRHR challenges among young people [42].

### Limitation and strengths of the study

This study provides a comprehensive analysis of the provincial case of multisectoral collaboration dynamics influencing the implementation of CSE in Eastern Province, Zambia. While it may not be possible to transfer the findings to the general pattern of collaboration dynamics across all the provinces, one should interpret them within this context, and we have made efforts to describe the context thoroughly so that the reader can judge the transferability of our findings to other contexts. We further documented the research context to help interested readers to assess the possibility of transferring the findings to similar contexts. The inclusion of national-level stakeholders from both government and private organisations facilitated an in-depth understanding of the pattern of collaboration dynamics lessons influencing the implementation of CSE in Zambia. This further enabled us to uncover specific knowledge on factors, challenges, and opportunities that affect effective implementation and integration of CSE in schools. Another strength of the study is the use of the theoretical framework to guide its development and implementation. The framework enabled us to analyse the rich data source and generate an in-depth understanding of the collaboration perspective. However, we limited the analysis to exploring collaboration dynamics, leaving out other crucial components of the framework such as actions and outcomes. These excluded parts of the framework will be explored fully in a separate study.

## Conclusion

This qualitative study explores the multisectoral collaboration dynamics influencing the implementation of Comprehensive Sexuality Education Framework at the provincial level in Zambia. The findings highlight the importance of mechanisms influencing principled engagement, shared motivation, and capacity for joint action among stakeholders to enhance the collaboration process in the delivery of CSE.

Our study shows that principled engagement is an important ingredient in shaping effective multisectoral collaboration in delivery of CSE programmes. Provincial leadership plays a significant role in facilitating the principled engagement and coordination of the CSE multisectoral activities. However, currently, provincial leadership has inadequate authority or power to compel stakeholders who are either not transparent and/or directly implementing activities without their involvement. Therefore, this suggests the government should reform the policy that mandates transparent budget declarations from all implementing actors to provincial authorities. This study also reveals several bottlenecks including exclusion and/or sidelining of some actors, financial constraints, and limited formal established relationships, which limit meaningful engagement in this collaboration process. At the same time, the government, through Ministries of Education and Health should strengthen the capacity of provincial leadership in collaborative governance of complex interventions like CSE and ensure that the leadership manages all the stakeholders and resources in a transparent manner.

This study further identified that having shared motivation among stakeholders was a major driver in shaping the collaboration trajectory. Stakeholders revealed that having a common desire to address several SRHR challenges was a key factor in propelling this collaboration forward. Furthermore, the ability of stakeholders to appreciate the different understanding of CSE and their willingness to give up what divides them was also a crucial factor that inspired them to collaborate. This shows that although social norms and values may negatively impact the implementation of CSE, constant engagement of all stakeholders is a crucial factor for discovering their felt needs, perspectives and position on the programmes. This consultative approach will go a long way toward achieving consensus and align the programme to the social context

Finally, this study also demonstrates the crucial role of strengthening provincial leadership capacity for joint action in collaborative delivery of CSE. The current disconnected collaboration in promoting child protection and addressing SRHR challenges including child marriages and pregnancies requires immediate transformative coordinated response. This also calls for strengthened multisectoral collaboration through provincial administration to discuss child protection and CSE-related issues with all the stakeholders in the provinces. These meetings would encourage stakeholders to appreciate the efforts of others, plan, collaborate and monitor the activities together. Future research using participatory and embedded approaches should be conducted to investigate collaboration dynamics and joint actions influencing the implementation of CSE from the district and community levels.

## Supplementary Material

Draft tools Mnauscript.docx

## Data Availability

The study data can be requested from the author.

## Abbreviations

AIDS: Acquired immune deficiency syndrome; CSE: Comprehensive Sexuality Education; Community Health Workers: CHWs; SRHR: Sexual Reproductive Health Rights; SDGs: Sustainable Development Goals; LMICs: low- and middle-income countries; UNICEF: United Nations Children’s Fund; UNESCO: United Nations Educational, Scientific and Cultural Organization; United Nations Population Fund: UNFPA; NGOs: non-governmental organization; MDGs: Millennium developmental goals; GBV: Gender-Based Violence; MoH: Ministry of Health; MoE: Ministry of Education.
