# Supplementary material for: Exploring multisectoral collaboration in implementing comprehensive sexuality education framework at the provincial level in Zambia: a qualitative study
Source: Glob Health Action. 2025 Aug 29;18(1):2547436. doi: 10.1080/16549716.2025.2547436 (PMC12404052; doi:10.1080/16549716.2025.2547436)
Supplement: Draft tools Mnauscript.docx [file ZGHA_A_2547436_SM7703.docx]

## Study Title: Exploring multisectoral collaboration in implementing Comprehensive Sexuality Education Framework at the provincial level in Zambia

## Introduction

1. How is your department involved in implementing CSE at the provincial level?
2. When did your department begin implementing CSE in the province?
3. Which other stakeholders or sectors do you collaborate with in implementing CSE (e.g., sensitisation, teacher training, service delivery)?

## Principled Engagement

1. How are different sectors and actors involved in delivering CSE at the provincial level?
2. How is CSE implementation managed across sectors in the province?

- Probe for availability of coordination structures such as provincial committees, intersectoral meetings, school assemblies, church sessions, etc.

1. Who are the key actors involved in the provincial coordination process, and what roles do they play?
2. What are the functions of the provincial committee or structure coordinating CSE?

- Probe for planning, policy direction, joint decision-making, and governance.

1. What are the benefits of having a multisectoral coordination committee at the provincial level?
2. What aspects of CSE coordination have worked well at the provincial level?
3. What challenges have been experienced in coordinating CSE across sectors in the province? How have these been addressed?
4. How can the engagement and collaboration process in implementing CSE at the provincial level be improved?

## Shared Motivation

1. What are the key issues motivating different sectors to collaborate on CSE implementation in the province?
2. Probe for shared concerns such as SRHR challenges, school dropouts, early marriages, or limited resources.
3. How motivated are provincial actors to address SRHR challenges through CSE? What drives their motivation?
4. What factors are affecting actors’ motivation to collaborate on CSE implementation at the provincial level?
5. What can be done to strengthen motivation and commitment among stakeholders for multisectoral collaboration in CSE?

## Capacity for Joint Action

1. What are the key CSE activities that stakeholders jointly implement at the provincial level?

- Examples of collaboration: teacher training, community sensitisation, campaigns against child marriage, re-entry policy implementation, service linkages, referrals, counselling, and SRHR service provision.

1. What has worked well in collective action for CSE at the provincial level?

- Probe for availability of resources, power dynamics, and enabling social norms.

1. What has not worked well in collective action for CSE at the provincial level?

- Probe for limitations in resources, power imbalances, or restrictive social norms.

1. What factors are limiting stakeholders’ ability and capacity to collaborate effectively at the provincial level?
2. What are the potential ways to strengthen joint action and multisectoral collaboration in CSE implementation?
3. Are there any risks or downsides to having too much collaboration among stakeholders?

## Conclusion

1. Is there anything else you would like to share that you feel is important for improving the CSE programme through multisectoral collaboration in Eastern Province?

Thank you so much for your participation.

# END OF INTERVIEW
